# Dissociation of the role of the prelimbic cortex in interval timing and resource allocation: beneficial effect of norepinephrine and dopamine reuptake inhibitor nomifensine on anxiety-inducing distraction

**DOI:** 10.3389/fnint.2012.00111

**Published:** 2012-12-03

**Authors:** Alexander R. Matthews, Olivia H. He, Mona Buhusi, Catalin V. Buhusi

**Affiliations:** ^1^Department of Psychology, USTAR BioInnovations Center, Utah State UniversityLogan, UT, USA; ^2^Department of Neurosciences, Medical University of South CarolinaCharleston, SC, USA

**Keywords:** affective disorder, dopamine, interval timing, nomifensine, norepinephrine

## Abstract

Emotional distracters impair cognitive function. Emotional processing is dysregulated in affective disorders such as depression, phobias, schizophrenia, and post-traumatic stress disorder (PTSD). Among the processes impaired by emotional distracters, and whose dysregulation is documented in affective disorders, is the ability to time in the seconds-to-minutes range, i.e., interval timing. Presentation of task-irrelevant distracters during a timing task results in a delay in responding suggesting a failure to maintain subjective time in working memory, possibly due to attentional and working memory resources being diverted away from timing, as proposed by the Relative Time-Sharing (RTS) model. We investigated the role of the prelimbic cortex in the detrimental effect of anxiety-inducing task-irrelevant distracters on the cognitive ability to keep track of time, using local infusions of norepinephrine and dopamine reuptake inhibitor (NDRI) nomifensine in a modified peak-interval procedure with neutral and anxiety-inducing distracters. Given that some anti-depressants have beneficial effects on attention and working memory, e.g., decreasing emotional response to negative events, we hypothesized that nomifensine would improve maintenance of information in working memory in trials with distracters, resulting in a decrease of the disruptive effect of emotional events on the timekeeping abilities. Our results revealed a dissociation of the effects of nomifensine infusion in prelimbic cortex between interval timing and resource allocation, and between neutral and anxiety-inducing distraction. Nomifensine was effective only during trials with distracters, but not during trials without distracters. Nomifensine reduced the detrimental effect of the distracters only when the distracters were anxiety-inducing, but not when they were neutral. Results are discussed in relation to the brain circuits involved in RTS of resources, and the pharmacological management of affective disorders.

## Introduction

**Figure 1:**
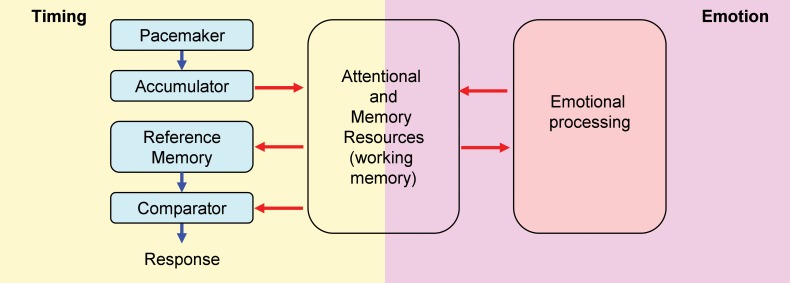
**Relative Time-Sharing model.** The Relative Time-Sharing model assumes that timing and emotional processing are concurrent processes which share working memory and attentional resources. Task-irrelevant emotional events are assumed to take away working memory and attentional resources from timing; without resources, timing is delayed. Since emotional events seem to have a sustained (long-lasting) effect on resource allocation, interval timing could be delayed considerably by emotional events (modified from Buhusi, 2012).

Attentional and working memory resources are crucial for the ability to keep track of time in the seconds-to-minutes range, i.e., interval timing (Buhusi and Meck, 2009a). The time keeping ability, and the effect of distracters on timing, can be tested within the Peak Interval (PI) procedure. Within the internal clock paradigm (Gibbon et al., 1984) (Figure 1, left panel), regular pulses emitted by a pacemaker, accumulate and are temporarily stored in working memory. Upon the subject being rewarded at the criterion (objective) time, the number of pulses in working memory (subjective time) is stored in reference memory. Responses are generated based on the ratio comparison between the number of pulses in working and reference memory. In trained subjects, responding is low at the beginning of the trial, reaches its peak about the time subjects are rewarded, and decreases afterward, when the current time (in working memory) is much larger than the criterion time (stored in reference memory). However, presentation of task-irrelevant distracters during the PI procedure results in a delay in responding (Buhusi and Meck, 2009a; Buhusi, 2012), suggesting a failure to maintain subjective time in working memory, possibly due to the attentional and working memory resources being diverted away from timing toward processing the distracter (Figure 1). According to this Relative Time-Sharing (RTS) model (Buhusi, 2003; Buhusi and Meck, 2009a), distracters result in a difference between the subjective (perceived) time and the objective time, thus explaining why “time flies when you are having fun,” but also how food gets burnt when little attention is paid to cooking.

Resource re-allocation is exacerbated by anxiety-inducing task-irrelevant distracters, resulting in impairing effects. For example, when asked to keep a face in working memory (primary task), the presentation of emotional faces (secondary task) impaired recognition memory (Dolcos and McCarthy, 2006). Indeed, the amygdala is strongly activated during emotional distracters and the dorso-lateral prefrontal cortex (dlPFC) is deactivated during emotional distracters (Dolcos and McCarthy, 2006; Pessoa, 2008; Denkova et al., 2010), thus decreasing resources allotted to the primary task. Similarly, the presentation of emotionally charged distracters during the uninterrupted to-be-timed signal results in a considerable delay in responding relative to neutral distracters (Aum et al., 2004; Brown et al., 2007). According to the RTS model, anxiety-inducing distracters divert attentional and working memory resources away from timing (Figure 1, left panel) to emotional processing (Figure 1, right panel) (Schirmer, 2011), thus reducing the ability to maintain the subjective time in working memory (Buhusi, 2003; Buhusi and Meck, 2009a). These task-irrelevant emotionally charged distracters would effectively keep timing “shut off” (time not stored in working memory) until emotional processing ceases (Schirmer, 2011), thus delaying timing for considerably longer durations than neutral distracters (Aum et al., 2004; Brown et al., 2007).

Conversely, when the emotional variable is embedded within the primary task, emotion enhances processing of the primary task. For example, when asked to discriminate between a long or a short presentation of an (emotional) face, human participants perceive angry emotional faces as being of longer duration than neutral faces (Droit-Volet and Meck, 2007), possibly due to re-allocation of resources toward the timed emotional stimulus: angry-looking faces increase amygdalar activation compared to happy faces (Davidson and Irwin, 1999), and may increase attention to the timed stimulus, thus facilitating time accumulation. In turn, selective attention to anxiety-inducing stimuli is abolished by inactivation of the amygdala, particularly baso-lateral amygdala (BLA), and may enhance time processing. For example, BLA lesions enhance the ability of rats to simultaneously time an aversive and an appetitive stimulus, due to the reduced fear disruption of dividing attention (Meck and Macdonald, 2007). Thus, emotion may have both impairing and enhancing effects on time processing (Macdonald and Meck, 2004; Droit-Volet and Meck, 2007; Etkin and Wager, 2007; Meck and Macdonald, 2007).

Learning and memory abilities are altered in patients with depression, PTSD, schizophrenia, and phobias (Davidson and Irwin, 1999; Rose and Ebmeier, 2006; Etkin and Wager, 2007; Gohier et al., 2009; Amir and Bomyea, 2011). A recent line of pharmacological treatment for these disorders involves norepinephrine and dopamine reuptake inhibitors (NDRIs), which indirectly increase neurotransmission in the noradrenergic (NE) and dopaminergic (DA) pathways. In turn, both DA and NE modulate the internal clock (Buhusi and Meck, 2010). DA agonists speed-up, and DA antagonists slow-down timing (Buhusi and Meck, 2002, 2005; Matell et al., 2004, 2006; Taylor et al., 2007; Coull et al., 2011). Moreover, NE modulates interval timing in both human participants (Rammsayer et al., 2001) and rodents (Penney et al., 1996). Nevertheless, the specific roles of DA and NE in interval timing at various brain sites are less understood.

Here we investigate the role of the prelimbic cortex in the detrimental effect of anxiety-inducing task-irrelevant distracters on the cognitive ability to keep track of time, in a modified version of the PI procedure with distracters (Buhusi and Meck, 2006), where emotionally charged auditory distracters were presented during the uninterrupted to-be-timed visual signal (Brown et al., 2007). As in previous studies (Aum et al., 2004; Brown et al., 2007), we expected the anxiety-inducing distracters to have detrimental effects on timing, and to delay responding much longer than when the distracters were neutral (Buhusi, 2012). We also hypothesized that interval timing and working memory for time depend on the NE and DA systems (Buhusi and Meck, 2002, 2005, 2009a; Coull et al., 2011). Therefore, local infusions of NDRI nomifensine in prelimbic cortex were expected to alter NE and DA transmission (Robinson and Wightman, 2004; Masana et al., 2011), and affect both clock speed (in trials without distracters) and maintenance of working memory for time (in trials with distracters). Given that some anti-depressants have beneficial effects on attention and working memory (Rammsayer et al., 2001), e.g., decreasing emotional response to negative events (Masana et al., 2011), we anticipated that nomifensine would improve maintenance of information in working memory in trials with distracters, resulting in a decrease in the disruptive effect of emotional events on the cognitive ability of timekeeping.

## Materials and methods

### Subjects

Twenty-two naïve Sprague-Dawley male rats, 300–350 g (3 months old at the beginning of the experiment) were housed individually in a temperature-controlled room, under a 12/12 h light-dark cycle, with water given *ad libitum*. Rats were maintained at 85% of their *ad libitum* weight by restricting access to food (Rodent Diet 5001, PMI Nutrition International, Inc., Brentwood, MO). All experimental procedures were conducted in accordance with the National Institutes of Health's Guide for the Care and Use of Laboratory Animals (1996).

### Apparatus

The apparatus consisted of 12 standard rat operant chambers (MED Associates, St. Albans, VT) housed in sound attenuating cubicles, of which four were used for fear conditioning and the other eight for interval timing. An auditory stimulus was first used during fear conditioning in the fear conditioning chambers, then later used as an anxiety-inducing distracter during the timing task in the timing chambers. The fear conditioning chambers and the interval timing chambers were made distinctive as follows: the fear conditioning chambers contained a dipper entry space for a liquid dipper (not used in the experiment); no lever was inserted in the boxes at any time; no food was given inside these chambers; pine pellets (Feline Pine Cat Litter, West Palm Beach, FL) were placed in the waste pan. In contrast, the interval timing chambers contained four nose pokes (not used in the experiment) and a lever; food was provided for lever-pressing at the right time; the bedding used in these boxes was cedar shavings (Grreat Choice, Phoenix, AZ).

In the fear conditioning chambers the grid floor was connected to shockers and scramblers controlled by a Med Associates interface, generating a 1 s 0.85 mA foot shock. The fear conditioning stimulus was an 85 dB white noise produced by a white-noise generator (MED Associates, St. Albans, VT). The intensity of the distracter was measured with a sound-level meter (Realistic Radio Shack, Model 33–2050) from the center of the silent box.

The interval timing chambers were equipped with a single fixed lever situated on the front wall of the chamber. According to the schedule, 45 mg precision food pellets (PMI Nutrition International, Inc., Brentwood, MO) were delivered in a food cup situated on the front wall, 1 cm above the grid floor, under the center lever, by a pellet dispenser. The to-be-timed visual stimulus was a 28 V 100 mA house light mounted at the center-top of the front wall. The auditory distracter was an 85 dB white noise produced by a white-noise generator (MED Associates, St. Albans, VT) mounted on the opposite wall from the response levers. A 66 dB background sound produced by a ventilation fan was present throughout the session.

### Behavioral training

For details of training and testing in the peak-interval timing procedure with distracters, see Buhusi and Meck (2006). For details of training and testing with emotional distracters in the peak-interval timing procedure, see Brown et al. (2007). Relevant details are given below.

### Fixed-interval (FI) training

All timing sessions were conducted in the eight timing chambers. After being shaped to lever press, rats received five daily sessions of fixed-interval (FI) training, during which the first lever press 40 s after the onset of the visual signal was reinforced by the delivery of a food pellet and turned off the house light for the duration of a random 120 ± 30 s inter-trial interval (ITI).

### Peak–interval (PI) training

Afterward, rats received five sessions of peak-interval training during which FI trials were randomly intermixed with non-reinforced PI trials in which the visual signal was presented for a duration three times longer than the FI, before being terminated irrespective of responding. Trials were separated by a 120 ± 30 s random ITIs.

### Fear conditioning

Rats were randomly assigned to two groups. Rats in the FEAR group were placed in the fear conditioning chambers, where they received six pairings of a 5 s white noise and a 1 s 0.85 mA foot shock, separated by random intervals (2–6 min long). Rats in the CTRL group were placed in the fear conditioning chambers for an equivalent amount of time, where they received six presentations of the 5 s white noise separated by random intervals (2–6 min long) (no foot shock). Rats received two 30 min sessions of fear conditioning, one before and one after the surgery (Brown et al., 2007).

### Surgery

During aseptic surgery under isoflurane anaesthesia, 26-gauge bilateral cannula guides (PlasticsOne, Roanoke, VA) were implanted aiming at the prelimbic cortex (AP 2.5 mm, ML ± 0.6 mm, DV −3.5 mm) (Paxinos and Watson, 1998) and embedded in dental cement. Rats were given at least 3 days to recover from surgery before retraining began again. Data (available, but not shown) indicated that rats responded reasonably well post-recovery. Rats were given six sessions of PI re-training before any local infusions began.

### Freezing behavior testing and re-training

Rats in the FEAR group were placed in the fear conditioning chambers where they received two presentations of the noise in extinction followed by two noise-shock pairings, at 4 min intervals. The session lasted 20 min. For rats in the CTRL group, the white noise stimulus was not paired with the foot shock. Behavior was recorded and freezing behavior was scored by two-independent observers in 2.5 s bins. The percent agreement score between the two observers was 89.64 ± 1.25 percent. Fear conditioning testing and re-training was followed by one session of PI re-training, as described above.

### Local infusions

Cannulae injectors aiming at mPFC were lowered into the cannula guides, extending 1 mm below the guides. Rats received intracranial injections of either saline or norepinephrine and dopamine reuptake inhibitor (NDRI) nomifensine (nomifensine maleate salt, Sigma Aldrich, St. Louis, MO), dissolved in 45% cyclodextrin (methyl-beta-cyclodextrin, Sigma Aldrich, St. Louis, MO). Rats received microinjections of 0.5 μL nomifensine solution (4 μg/side) or saline, bilaterally, at a rate of 0.25 μL/min, over 2 min, followed by a 2 min interval to allow the drug to infuse the tissue. Five minutes afterward, rats were placed into the timing chambers for testing in a timing sessions with noise (see next paragraph). Infusion sessions were separated by three no-drug sessions as follows: one post-drug PI re-training session, one fear conditioning testing and re-training session, and one post-fear conditioning PI re-training session. The order of drug infusion (saline or nomifensine) was counterbalanced between animals.

### Timing sessions with noise and drug infusion

Five minutes after drug infusion, rats received two consecutive 1.5 h sessions of interval timing testing, during which rats received 20 FI and 14 PI trials randomly intermixed with 6 PI trials with noise (PI + N). PI + N trials were similar to PI trials, except that the 5 s white noise was presented (during the uninterrupted visual to-be-timed stimulus), 5 s from the onset of the trial.

### Histology

**Figure 2:**
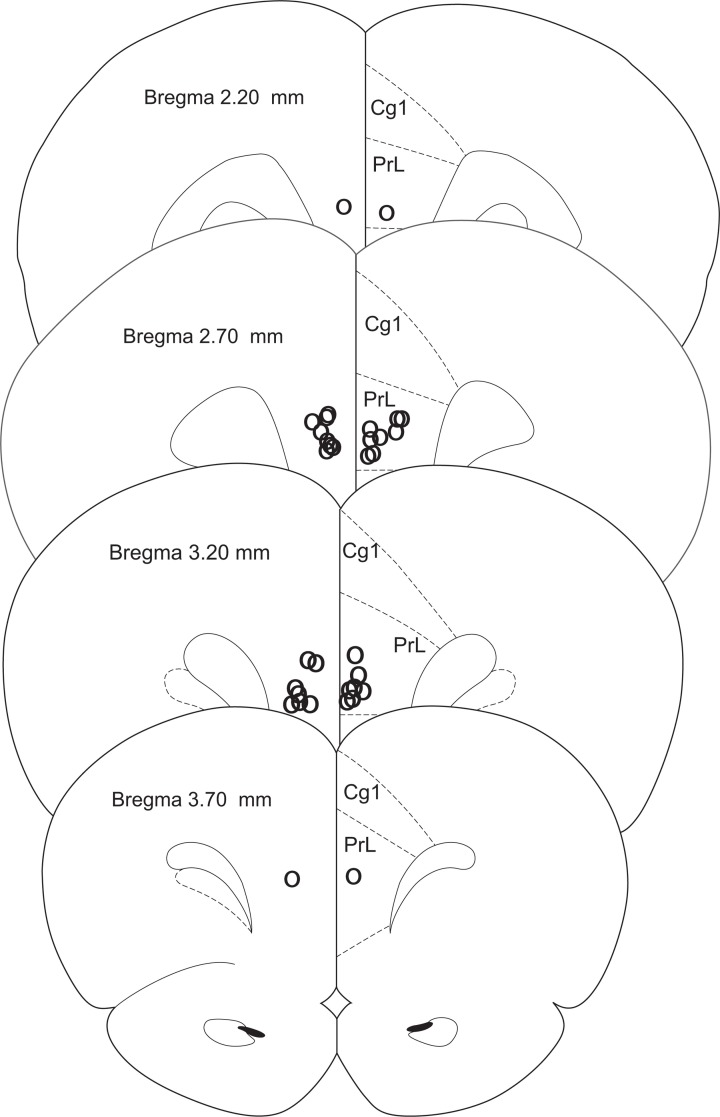
**Histology.** Cannula placements in the present experiment. Only rats with injections in the prelimbic cortex were used in the experiment (CTRL *n* = 6, FEAR *n* = 11).

Rats were anesthetized with isoflurane overdose and transcardially perfused with formalin; their brains were collected and sectioned on a vibratome. Sixty-micron sections were placed on slides and stained with sky-blue for histological analyses. Three rats were eliminated due to incorrect cannula placement; two rats lost their cannulae before testing was completed and were eliminated from the study (CTRL *n* = 6, FEAR *n* = 11) (Figure 2).

### Data collection and analysis

The experimental procedures were controlled through a MED Associates interface connected to an IBM-PC compatible computer running a MED-PC software system (MED Associates, 1999). Lever presses were recorded in real time. Lever presses from PI and PI + N trials from the first 1.5 h test session with drug infusion were used to estimate the peak time, peak rate, and precision of timing (width of the response functions) for each rat. The number of responses (in 4 s bins) was averaged daily over trials, to obtain a mean response rate function for each rat. Analyses were conducted on the data from a 100 s interval-of-interest starting at the onset (for PI trials) or 20 s from the onset of the to-be-timed signal (for PI + N trials). The average response rate in the interval-of-interest was fit using the Marquardt-Levenberg iterative algorithm (Marquardt, 1963) to find the coefficients (parameters) of a Gaussian + linear equation that gave the best fit (least squares minimization) between the equation and the data (Buhusi and Meck, 2000). The algorithm provided the following parameters of the response curve: the accuracy of timing (peak time), precision of timing (width of response function), and peak rate of response (for details see Buhusi and Meck, 2000).

To further investigate the effect of the presentation of the noise and the effect of the drug on the dynamics of timing behavior, individual-trial analyses were performed as described in (Church et al., 1994; Swearingen and Buhusi, 2010). Briefly, during individual trials, the distribution of lever presses can be approximated by a low-high-low function. Analysis algorithms described in (Swearingen and Buhusi, 2010) were used to extract the *start* and *stop* times during individual trials. The *start* time is the time point at which there is a significant increase in response rate during the trial (at the transition from the low to high states). The *stop* time is the point during the trial at which there is a significant decrease in response rate (at the transition from the high to low states). Trials without temporal control (about 20% of total trials) were eliminated from individual-trial analyses based on the very conservative criteria defined in (Church et al., 1994), except for PI + N trials in the FEAR group: to accommodate for the disruption in response caused by the presentation of the noise, in PI + N trials in the FEAR group analyses were conducted on data after the noise [in the interval-of-interest (20–120 s), same interval as for the curve fitting analysis, see above] and there were no exclusion criteria for *start* time.

The dependent variables *peak time*, *width of function*, *start time*, *stop time*, and *the coefficient of variation (CV) of the start and stop times* were submitted to mixed ANOVAs with independent between-subject variable *group* (FEAR, CTRL) and within-subject variables *trial type* (PI, PI + N) and *drug* (SAL, NOM), followed by planned comparisons. Statistical tests were evaluated at a significance level of 0.05.

## Results

### Long-lasting emotional response to the fear-inducing event

**Figure 3:**
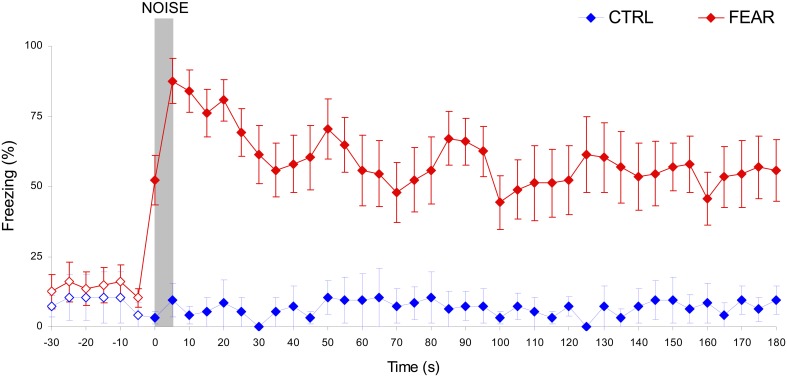
**Long-lasting freezing behavior following the presentation of the auditory distracter in the FEAR, but not CTRL rats.** Average percent freezing behavior (±SEM) in the fear conditioning context during freezing behavior testing and re-training sessions, before, during, and after the presentation of the noise (in extinction, no shock). Unlike CTRL rats, FEAR rats show reliable, long-lasting freezing behavior after, but not before the presentation of the noise. Empty symbols show time where emotional response (freezing behavior) did not differ reliably between FEAR and CTRL rats, *p* > 0.05. The gray bar indicates the presentation of the auditory distracter.

Rats' emotional response in the fear context before and after the noise was measured during freezing behavior testing and re-training sessions, as shown in Figure 3. Before the noise occurred, both the CTRL and FEAR groups show similar low levels of freezing, all *t*s_(15)_ < 1.62, *p* > 0.12. Moreover, no freezing behavior was shown after the noise when the noise was not paired with the foot-shock (CTRL rats), all *t*s_(5)_ < 1.19, *p* > 0.14. In contrast, when the noise was emotionally charged by being paired with foot shock (FEAR group), rats showed reliable freezing behavior following the presentation of the noise in extinction (without shock presentation), all *t*s_(15)_ > 3.98, *p* < 0.001. Interestingly, the strong levels of freezing (e.g., 87.5% during the noise) lasted for several minutes after the noise ended (see Figure 3), and decreased slowly to baseline levels before the next presentation of the noise. This long-lasting effect of the presentation of the emotionally charged event (FEAR group), explains the considerable delay in timing by the presentation of the same fear-inducing event in the timing context, see below.

### No effect of treatment on variability of timing (width of the response function)

**Figure 4:**
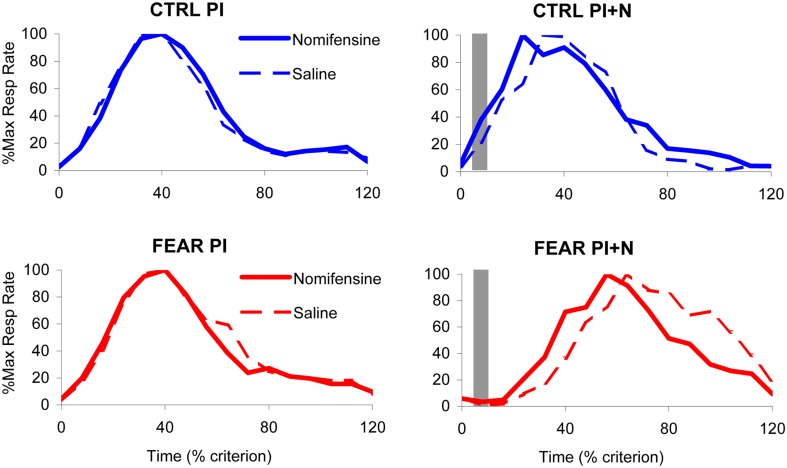
**Effect of nomifensine infusion on average timing functions.** Average maximum percent response (lever pressing) rate in rats trained to time a 40 s criterion signaled by a visual stimulus when presented with a neutral distracter (CTRL group, *upper panels*) or an emotionally charged distracter (noise previously paired with foot shock, FEAR group, *lower panels*). *Left panels*: Peak interval (PI) trials (without noise): nomifensine has no reliable effects in either group. *Right panels*: Peak interval with noise (PI + N) trials (with noise): when emotionally charged (FEAR group), the distracter shifts the response function rightward considerably relative to neutral events (CTRL); nomifensine reduces the delaying effect of the distracter only when the distracter is emotionally charged (FEAR group). The gray bars indicate the presentation of the auditory distracters.

The average maximum percent response rate functions in PI and PI + N trials, with and without auditory distracter are shown in Figure 4. These results suggest that, the variability in timing (width of the timing function) is not affected by either treatment. Indeed, a mixed ANOVA of the width of the timing functions with between-subject variable group (FEAR, CTRL) and within-group variables drug (SAL, NOM) and trial type (PI, PI + N), failed to indicate any reliable main effects or interactions, all *F*s_(1, 15)_ < 0.95, *p* > 0.35, suggesting that neither nomifensine nor the distracter had any reliable effects on variability of response in either group. In short, the treatments simply shifted the timing functions without changing their width. Therefore, for the remainder of the paper, we will focus only on the effect of treatment on timing (i.e., on the peak time).

### No effect of nomifensine on timing in PI trials (without distracter)

The average maximum percent response rate functions in PI trials (without auditory distracter) are shown in the left panels of Figure 4. Under saline, the PI timing functions peaked at 36.51 ± 2.21 s in FEAR rats, and at 35.25 ± 1.46 s in CTRL rats. Under nomifensine, the PI timing functions peaked at 34.69 ± 1.39 s in FEAR rats, and at 36.92 ± 2.34 s in CTRL rats, suggesting that nomifensine had no specific effects relative to saline. Although reliably lower than 40 s for both saline and nomifensine, all *t*s > 2.63, *p* < 0.05, the estimated peak times were relatively close to the criterion time, indicating that rats acquired the timing task. Indeed, a mixed ANOVA of peak time with between-subject variable group (FEAR, CTRL) and within-group variable drug (SAL, NOM) failed to indicate reliable effects of group, drug, or interactions, all *F*s_(1, 15)_ < 0.73, *p* > 0.41, suggesting that nomifensine had no reliable effects in trials without noise distracter (PI trials) in either group.

### The auditory distracter delays timing only when it is fear-inducing

The top-right panel of Figure 4 indicates that the presentation of the noise has no effect on timing when the distracter was neutral (CTRL group). The PI + N timing functions peaked at 38.42 ± 3.38 s under saline, and at 32.44 ± 3.61 s under nomifensine, not significantly different from the 40 s criterion, all *t*s < 2.09, *p* > 0.05. In contrast, as seen in the bottom-right panel of Figure 4, responding was considerably delayed by the presentation of the fear-inducing distracter under both saline and nomifensine, relative to trials without the distracter. In the FEAR group, the PI + N timing functions peaked at 69.77 ± 5.50 s under saline, and at 56.83 ± 3.19 s, under nomifensine. The difference between groups was confirmed by a mixed ANOVA of peak time in PI + N trials, with between-subject variable group (FEAR, CTRL) and within-group variable drug (SAL, NOM) which indicated a reliable main effect of group, *F*_(1, 15)_ = 38.0, *p* < 0.001, suggesting that the distracter has a reliably different effect when it is emotionally charged or neutral.

**Figure 5:**
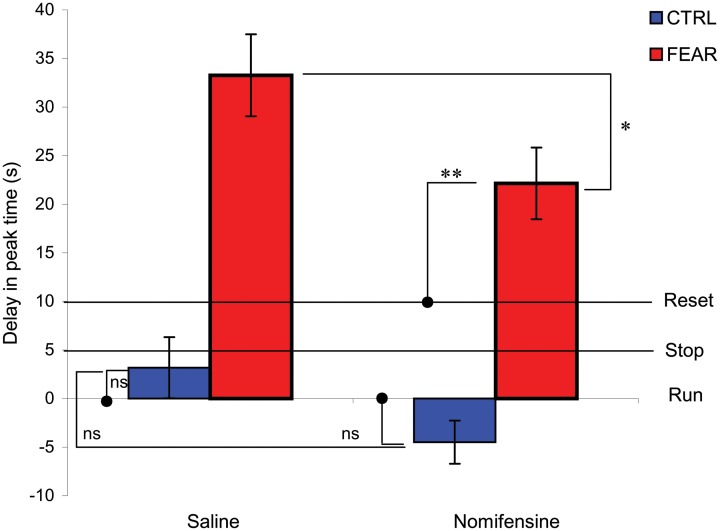
**Average delay for trials with and without noise distracter.** Average peak time delay (±SEM) in trials with distracters (PI + N) relative to trials without distracters (PI). Should the distracter have no effect, the clock would continue to time (run, delay = 0 s). Should the clock stop timing during the distracter, the delay would be equal to the duration of the noise (stop, delay = 5 s). Should the clock restart timing after the distracter, the delay would be equal to the duration of the noise, 5 s, plus the duration of the pre-distracter interval, 5 s (reset, delay = 10 s). Rats ignored a neutral distracter (CTRL). In contrast, rats over-reset when the distracter is emotionally charged (FEAR). Nomifensine reliably reduces the time delay. ^*^p < 0.05; ^**^p < 0.01; ns, *p* > 0.05.

To further investigate the differential effect of nomifensine in the two groups in trials with distracters, relative to trials without distracters, we computed and analyzed a delay in peak time between the two trial types, as shown in Figure 5. Should the distracter have no effect, the rats would continue to time (run, delay = 0 s). Should rats stop timing during the distracter, the delay would be equal to the duration of the noise (stop, delay = 5 s). Should rats restart timing after the distracter, the delay would be approximately equal to the duration of the noise, 5 s, plus the duration of the pre-distracter interval, 5 s (reset, delay = 10 s). Results indicate no delay (run) in the CTRL group, *t*_(5)_ = 1.01, *p* > 0.36, but a considerable delay by an emotionally charged noise (FEAR group), beyond a full reset of timing. Indeed, a mixed ANOVA with between-subject variable group (FEAR, CTRL) and within-subject variable drug (SAL, NOM) indicated a reliable effect of group, *F*_(1, 15)_ = 43.88, *p* < 0.001, drug *F*_(1, 15)_ = 5.83, *p* < 0.03, but no drug × group interaction *F*_(1, 15)_ = 0.20, *p* < 0.66. In the CTRL group, the neutral noise had no effect on delay (run), *t*_(5)_ = 2.01, *p* > 0.1. In contrast, in the FEAR group, the emotionally charged noise significant delayed timing more than the reset (over-reset), *t*_(10)_ = 5.52, *p* < 0.001. These results are consistent with those of Brown et al. (2007) and Aum et al. (2004), where an emotionally charged distracter resulted in an over-reset of timing.

### Nomifensine infusion reduces the delay in responding for fear-inducing distracters, but not for neutral distracters

The data from Figure 5 also indicates that nomifensine reduces the delay in peak time only when the noise is emotionally charged (FEAR group). A mixed ANOVA of the delay time between PI + N and PI trials with between-subject variable group (FEAR, CTRL) and within-subject variable drug (SAL, NOM), indicated a reliable effect of drug, *F*_(1, 15)_ = 5.83, *p* < 0.05 (see Figure 5). Planned comparisons indicated that in PI + N trials, nomifensine reliably decreases the delay in timing for the FEAR group, *F*_(1, 15)_ = 5.80, *p* < 0.05, but not in the CTRL group, *F*_(1, 15)_ = 1.49, *p* > 0.05. Moreover, in the FEAR group, the delay under nomifensine is reliably smaller than under saline, yet larger than reset, *t*_(10)_ = 3.29, *p* < 0.01.

### No effect of nomifensine on start and stop times in PI trials (without distracter)

**Figure 6:**
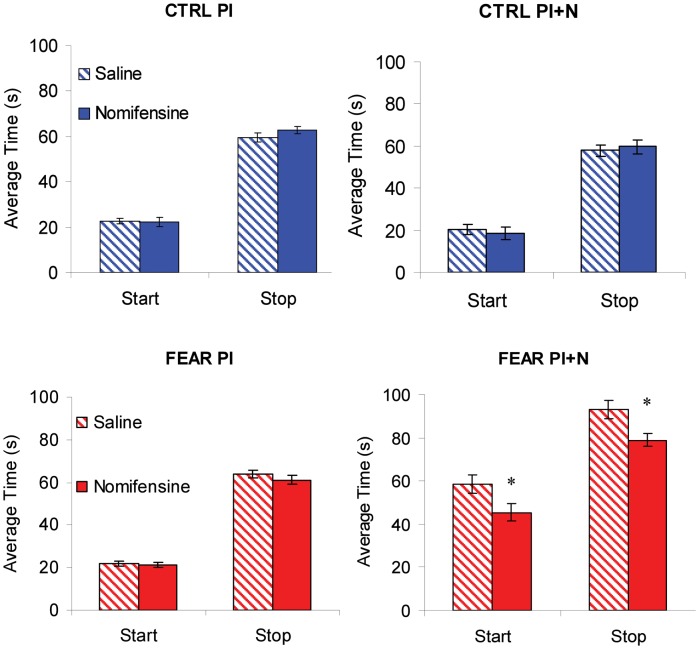
**Individual-trial dynamics.** Average estimated start and stop times (±SEM) in individual trials. Nomifensine reliably reduced both start and stop times only in trials with distracter (PI + N) and only when the noise distracter is emotionally charged (FEAR rats). ^*^p < 0.05.

To further investigate the effect of nomifensine on the dynamics of responding (lever pressing) in trials with and without noise, we followed the observation that during individual trials the rate of lever pressing has a low-high-low profile (Church et al., 1994). Using algorithms described in Swearingen and Buhusi (2010), we extracted and analyzed the *start* and *stop* times during individual trials. Briefly, the *start* time is the time of transitioning between the low and high state, and the *stop* time is the time of transitioning between the high and low state, in that individual trial. The start and stop times in PI trials (without noise) are shown in the left panels of Figure 6, suggesting that there was no effect of nomifensine on start and stop times in PI trials. The lack of effect of nomifensine on start and stop times is PI trials was confirmed by mixed ANOVAs with between-subject variable group (FEAR, CTRL), and within-subject variable drug (SAL, NOM), which failed to indicate reliable effects of group, drug, or interactions, all *F*s_(1, 15)_ < 1.73, *p* > 0.21 (see Figure 6, left panels).

The lack of effect of nomifensine in trials without noise (PI) may have been due to large variations in response, for example, in trials before and after trials with auditory distracter (PI + N). Considering the relatively long-lasting freezing behavior following the presentation of the noise (see Figure 3), rats were expected to have large disruptions in response immediately after a PI + N trial, but recover before the next PI + N trial. These differences in responding before and after a PI + N trial may have resulted in large variations in response, which may have obscured the effect of the drug in PI trials. Therefore, we extracted and contrasted the start and stop times in PI trials before and after PI + N trials, and their coefficients of variation. However, analyses of start and stop times and their coefficients of variation failed to indicate main effects of the group, all *F*s_(1, 15)_ < 2.31, *p* > 0.15, the before-after condition, all *F*s_(1, 15)_ < 3.24, *p* > 0.11, drug, all *F*s_(1, 15)_ < 0.45, *p* > 0.51, or interactions, all *F*s_(1, 15)_ < 2.54, *p* > 0.13, suggesting that the response in PI trials were relatively stable before and after a PI + N trial. Thus, the lack of effect of nomifensine in PI trials does not seem to be due to interference from trials with distracters.

### Nomifensine shortens both start and stop times after fear-inducing distracters, but not after neutral distracters

Mixed ANOVAs of start and stop times with between-subject variable group (FEAR, CTRL), and within-subject variable drug (SAL, NOM), indicated a reliable main effect of group, *F*s_(1, 15)_ > 58.49, *p* < 0.001. Planned comparisons indicated a reliable effect of nomifensine on the start and stop times in the FEAR group, *F*s_(1, 15)_ > 6.66, *p* < 0.02, but not in the CTRL group, *F*s_(1, 15)_ < 0.09, *p* > 0.77, suggesting that infusion of nomifensine resulted in a reliable decrease in both start and stop times only in FEAR rats (see Figure 6, right panels).

## Discussion

This study was aimed at elucidating both the impact of fear-inducing task-irrelevant distracters on interval timing, and the role of the NE and DA modulation of the prelimbic cortex in emotional processing of timed events. Our results suggest a dissociation of the effects of nomifensine in the prelimbic cortex on interval timing, explained by resource allocation (Relative Time-Sharing), after fear-inducing distraction, but not after neutral distraction. Rats ignore low-salience neutral distracters (run), stop timing during the medium-salience neutral distracters, and reset (restart timing from the beginning) after high-salience neutral distracters (Buhusi and Meck, 2000, 2006; Buhusi, 2012). Accordingly, the RTS model proposes that during neutral distracters, working memory for time decreases at a rate proportional to the salience of the distracter (Buhusi, 2003, 2012; Buhusi and Meck, 2009a). While some emotional distracters have transient (short-lived) effects, like neutral distracters (Droit-Volet and Meck, 2007), in our setting, the fear-inducing distracters may have been very salient, because they considerably delayed responding, beyond the reset (Aum et al., 2004; Brown et al., 2007), suggesting that resources are not returned back to timing until long after the distracter has ended. This “post-cue” effect (Aum et al., 2004; Brown et al., 2007) is consistent with our finding that rats in the FEAR group show a long-lasting emotional response long after the offset of the auditory distracter. However, under nomifensine, the delay in responding was shortened, and rats started and stopped timing earlier, suggesting that nomifensine decreased the fear-inducing effect of the distracter, and facilitated the return of resources from emotional processing back to interval timing.

Interestingly, nomifensine was effective only during trials with distracters (PI + N), but not during trials without distracters (PI trials), suggesting that at the current dose (4 μg/side) the drug does not change the speed of an internal clock. This finding is rather surprising, considering the strong DA modulation by nomifensine, and the putative role of DA in the control of the speed of an internal clock (Buhusi and Meck, 2010). Striatal infusion of nomifensine elevates DA release, with effects on operant behavior (Robinson and Wightman, 2004). Also, NE drugs are thought to selectively enhance mesocortical DA, due to the co-release of NE and DA at NE terminals (Masana et al., 2011). In addition, NE transporter (NET) is not specific for NE, and allows reuptake of DA as well. Therefore, blockage of both DAT and NET by NDRIs allows for a much higher amount of DA and NE to be released in medial prefrontal cortex (mPFC) (Masana et al., 2011). Since systemic administration of DA drugs alters the speed of an internal clock (Buhusi and Meck, 2005; Coull et al., 2011), we expected that increasing DA availability by nomifensine infusion to speed-up timing in PI trials, but this was not the case in our study.

One possible explanation for the ineffectiveness of nomifensine on the speed of an internal clock (in PI trials) is the increased variation in response. For example, considering the relatively long-lasting emotional response following the presentation of the noise, rats could have had large disruptions in response in PI trials that immediately follow a PI + N trial, but may have recovered before the next PI + N trial. This hypothesis was not supported by individual-trial analyses which failed to indicate differences in start and stop times, and in their CVs, in PI trials before and after PI + N trials. This finding mirrors that of Brown et al. (2007), which found that the delaying effect of the distracter is limited to trials with distracters, and does not “spill” into PI trials.

Nomifensine administration in mPFC was only effective in distracter (PI + N) trials, and only when the distracter was fear-inducing. Indeed, fear-inducing events have detrimental effects on cognition, in accord with reallocation of resources between the task at hand (e.g., interval timing) and emotional processing. Emotionally charged events create a markedly different pattern of activation within the ventral “emotional” and dorsal “executive” systems (Davidson and Irwin, 1999; Dolcos and McCarthy, 2006; Pessoa, 2008; Etkin et al., 2011; Johnson et al., 2011). Both fMRI studies in human participants and lesion studies in rodents indicate that emotional distracters produce working memory deficits by altering the relative activity of the dorsal and ventral systems (Dolcos and McCarthy, 2006; Denkova et al., 2010). The dorsal executive system is activated in working memory tasks (such as interval timing) (Buhusi and Meck, 2005; Pessoa, 2008). Impairing the function of the dorsal executive system results in working memory deficits in both human participants (Dolcos and McCarthy, 2006) and rodents (Kim et al., 2009). For example, working memory performance is hindered in human participants with decreased activation in dorsomedial and dorsolateral PFC (Pessoa, 2008; Denkova et al., 2010). Similarly, deficits in interval timing (which relies on working memory) have been reported in rodents following temporary inactivation of mPFC (Kim et al., 2009).

The dlPFC/mPFC is a “connectivity hub” which integrates motivation and cognitive executive functions (Chiew and Braver, 2011), and where cognition and emotion interact (Pessoa, 2008). Lesion studies indicate the importance of the frontal cortex for both interval timing (Kim et al., 2009) and emotion (Sierra-Mercado et al., 2011): rats with cortical lesions are unable to simultaneously attend to two concurrent intervals being timed (Olton et al., 1988). A similar effect was seen in rats presented with emotional stimuli during timing; the disruption was blocked by bilateral lesions of the amygdala (Meck and Macdonald, 2007). On the other hand, emotional information reaches amygdala by two pathways, a rapid but imprecise subcortical “low road,” and a slower cortical “high road” which provides more elaborate cognitive influences to be placed over emotional action (Ledoux, 2007; Johnson et al., 2011). The two tracts would act as two different mechanisms differentially activated by the particular arousal level (Droit-Volet and Meck, 2007; Chiew and Braver, 2011). A low arousal level would indicate attentional control; a high arousal level would indicate that motivational-survival systems are controlling behavior automatically. A high arousal level would increase the activation of the autonomic nervous system, which is associated with increases in clock speed (Droit-Volet and Meck, 2007). The two parallel tracts allow for the modulation of activity in the amygdala and allow cognitive influences to be placed over emotional action. The dlPFC/mPFC might work as a mediator between the frontal executive functions and the amygdalar emotional responses (Pessoa, 2008; Etkin et al., 2011).

Norepinephrine and dopamine may shift the balance between the cortical “high road” and the subcortical “low road,” by acting both in the amygdala and at cortical level, in opposite directions. Fear conditioning generates stress and is associated with NE reduction in the amygdala. NE reduction reduces the feedback within the amygdala and creates an overactive fear response (Johnson et al., 2011). In contrast, combined systemic administration of NET blocker reboxetine and NE alpha-2 blocker mirtazapine decrease fear and increase DA release in mPFC, thus providing a balancing mechanism to exert cognitive influences over emotional responses (Masana et al., 2012) … Similarly, DA receptor activation in amygdala removes its mPFC suppression (i.e., hypoactivity) (Rosenkranz and Grace, 2002), which increases the emotional response. In contrast, mPFC 6-OHDA lesions delayed extinction of fear, suggesting that mPFC DA modulates the response to fear-inducing cues, as in our experimental setting (Morrow et al., 1999). Therefore, the “high” pathway creates the ability to overcome emotional memories and to consciously balance the emotional response to the “low” pathway (Rosenkranz and Grace, 2002; Meck and Macdonald, 2007). Indeed, our experiment indicates that nomifensine's modulation of NE and DA cortical activity could offset the increased fear caused by the distracter, possibly by activating the cortical “high” road and reducing fear, thus decreasing both the start and stop in responding.

**Figure 7:**
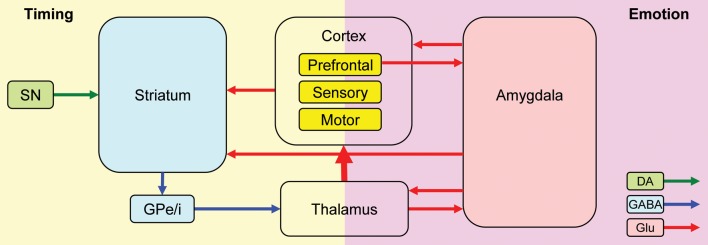
**A neurobiological RTS model: Interactions between timing and emotion circuits.** Interval timing relies on fronto-striatal circuits and cortical regions involved in attention and working memory. Emotional processing relies on direct thalamo-amygdalar projections (low road), and indirect thalamo-cortico-amygdalar projections (high road). Interactions between the timing and emotion circuits may occur at cortical level, or through direct amygdala-striatum projections. SNc, substantia nigra pars compacta; GPe/i, globus pallidus external/internal; BLA, basolateral amygdala; CeA, central nucleus of the amygdala; DA, dopamine; Glu, glutamate; GABA, gamma-aminobutyric acid. This simplified model of the timing–emotion interaction has been modified from Buhusi and Meck (2005) and Rosenkranz and Grace (2002).

Nomifensine within the prelimbic cortex may have altered the sharing of resources between timing and emotional processing. Recent explanations of attentional effects in interval timing, particularly in regard to the effect of task-irrelevant distracters (either neutral or emotionally charged), are done within the framework of the RTS model (Buhusi and Meck, 2006, 2009a; Buhusi, 2012) which assumes that distracters result in the reallocation of the limited pool of attentional and working memory resources from timing toward other processes (e.g., emotional processing) (Buhusi and Meck, 2009b; Schirmer, 2011; Buhusi, 2012). The model is compatible with the current understanding of the circuits involved in interval timing and emotional processing, and involves homologous relationships in humans and rodents (Uylings et al., 2003; Vertes, 2004) (Figure 7). Interval timing engages fronto-striatal functional circuits (Buhusi and Meck, 2005, 2009a) dependent on brain regions known to be involved in working memory, such as dlPFC in human participants (Stevens et al., 2007), and the mPFC in rodents (Kim et al., 2009) (Figure 7, left panel). Homologous relationships between the primate dlPFC and rodent mPFC have been also shown in regard to amygdalar connectivity, which is crucial for emotional processing (Sierra-Mercado et al., 2011) (Figure 7, right panel). The sharing of brain regions involved in working memory, between the circuits involved in timing (Figure 7, left) and emotional processing (Figure 7, right) provides support for a neurobiological RTS model, by which resource allocation is dependent on the modulation of activity in brain regions dealing with working memory (dlPFC for humans, mPFC for rodents) by both the circuits involved in timing and other processing (e.g., emotional). During timing, fronto-striatal circuits would engage working memory (dlPFC/mPFC). However, presentation of an emotionally charged distracter would also activate the amygdala, which would engage the dlPFC/mPFC in emotional processing, thus decreasing the relative activation on the fronto-striatal timing circuits. When emotional processing ceases (which could be long after the offset of the distracter), the activation of dlPFC/mPFC by (emotional) amygdalar circuits would decrease relative to their activation by fronto-striatal (timing) circuits, thus re-starting the timing process. In this framework, nomifensine would modulate activity within mPFC, to either decrease the fear-inducing effect of the distracter, and/or to reallocate resources toward timing, e.g., by increasing maintenance of temporal information in working memory. This may explain the “over-resetting” effect of the distracter, and the fact that nomifensine was effective only in PI + N trials, and only when distracters were fear-inducing.

On the other hand, results are inconsistent with an “attentional switch” or “flickering switch” (FS) model (Gibbon et al., 1984). Early explanations of attentional effects on interval timing have suggested an attentional switch in between the pacemaker and the accumulator, controlled by the presence and the salience of the to-be-timed signal (Gibbon et al., 1984), and dependent on the DA (Buhusi, 2003) and NE systems (Penney et al., 1996). Manipulations that affect the attention paid to timing were assumed to affect the latency to open/close the (flickering) switch, such that pulses from the pacemaker do not reach the accumulator, resulting in delayed responding (Lejeune, 1998; Zakay, 2000; Buhusi and Meck, 2002, 2005, 2009b). The FS model cannot address our findings. First, the switch is supposed to be closed during the uninterrupted presentation of the to-be-timed signal (Church, 1978, 1984), such that it cannot address the delaying of timing in our study (since the to-be-timed visual signal was not interrupted in PI + N trials), let alone the “over-reset” seen under saline in FEAR rats. Second, FS and RTS differ on both the location of the putative mechanism, before and after the accumulator respectively, and on the duration of action, either strictly during the interrupting event or throughout the task (see Buhusi and Meck, 2006). The FS switch is flickering only during the interrupting event, and is located before the accumulator; therefore, it would at best predict a stop (no pulses accumulated.) On the other hand, RTS is active throughout the task, not only during the noise but possibly after the noise as well, and it is located after the accumulator, at the level of working memory; therefore, RTS can explain the over-reset behavior in the present experiment. Thirdly, RTS is concurrent with timing (involves competition between the timer and other processes outside of the timer) while FS is a process inside the timer. Therefore, FS cannot predict an over-reset (since the timer itself cannot over-reset) while RTS is free from this restriction.

Moreover, while FS is a cognitive construct, RTS can been applied (as done in this paper) to neurobiological data (e.g., Buhusi and Meck, 2002, 2009a), as follows: first, past studies using retention intervals (gaps) indicated that DA has dissociable effects on the clock speed (in PI trials) and RTS (in trials with distracters) (Buhusi and Meck, 2002, 2007): systemic administration of DA agonists increases clock speed in PI trials and delays timing in trials with gaps; In contrast, despite being an indirect DA agonist, nomifensine had no effects in PI trials, and reduced the delay in timing in trials with distracters, suggesting that nomifensine infusions into mPFC affect RTS, but not clock speed, and that clock speed may rely on DA flow in other brain regions beside mPFC. Second, systemic administration of clonidine, an NE α2 agonist, results in delayed responding in PI trials (Penney et al., 1996), consistent with an increase in the latency of the FS to open. In contrast, in our study, nomifensine, an indirect NE agonist had no effects in PI trials, suggesting that Penney et al.'s FS interpretation of NE results is questionable. Finally, in our study nomifensine was effective only in trials with an fear-inducing distracter, but not when the distracter was neutral, which is consistent with the use of nomifensine in the treatment of depression and anxiety (Tejani-Butt et al., 2003; Jiao et al., 2006). These results can be easily addressed by the RTS model (Buhusi and Meck, 2006, 2009a; Buhusi, 2012), in that nomifensine decreases the fear-inducing effect of the distracter, and/or affects reallocation of resources toward timing, e.g., by increasing maintenance of temporal information in working memory. Therefore, nomifensine treatment may be beneficial in disorders characterized by impaired working memory processing, especially in affective disorders.

Indeed, emotional processing is dysregulated—either impaired or enhanced—in disorders such as schizophrenia, depression, phobias, and post-traumatic stress disorder (PTSD) (DSM-IV-TR, 2000). Alterations in the dorsal system involved in executive processing and ventral systems involved in emotional processing are reported in patients diagnosed as being depressed (Dolcos and McCarthy, 2006). Damage to the left dlPFC increases the likelihood of becoming depressed, while damage to the right dlPFC is linked to working memory impairments (Davidson and Irwin, 1999). Anxiety disorders, such as PTSD, are characterized by a specific deficiency in the ability to extinguish fear responses (Droit-Volet and Meck, 2007; Denkova et al., 2010; Sierra-Mercado et al., 2011), and by reoccurring and intrusive memories of the traumatic event. Extinction learning is hindered if the fronto-amygdalar circuit is dysregulated. PFC hypoactivity (Etkin and Wager, 2007; Etkin et al., 2011), and significant amygdalar activation (Davidson and Irwin, 1999) contribute to the emotional dysregulation in PTSD. Amygdalar hyperactivity is also seen in patients with social anxiety disorder and specific phobias (Rosenkranz and Grace, 2002). Moreover, heightened amygdalar activity can result in emotional responses to non-salient sensory stimuli, which in the absence of PFC-suppression, might lead to paranoia-like feelings (Rosenkranz and Grace, 2002). The dorsal system could modulate activity between emotional judgment and attention (Johnson et al., 2011). Greater control over fear and anxiety could be obtained in patients by increasing the PFC regulation of emotional events, for example, by reappraisal treatment, shown to activate the mPFC and to decrease negative emotions (Etkin et al., 2011). Solutions that allow for coping with emotional distractions in the executive and cognitively controlled frontal regions of the brain can help control emotional distraction (Denkova et al., 2010). Controlling mPFC activity, for example by NRDI antidepressants like nomifensine, may regulate working memory, and increase the quality of life for patients with affective disorders (Etkin and Wager, 2007).

### Conflict of interest statement

The authors declare that the research was conducted in the absence of any commercial or financial relationships that could be construed as a potential conflict of interest.

## References

[B1] Amir N. Bomyea J. (2011). Working memory capacity in generalized social phobia. J. Abnorm. Psychol. 120, 504–509 10.1037/a0022849 21381805 PMC3229920

[B3] Aum S. W. Brown B. L. Hemmes N. S. (2004). The effects of concurrent task and gap events on peak time in the peak procedure. Behav. Processes 65, 43–56 10.1016/j.beproc.2006.11.001 14744546

[B4] Brown B. L. Richer P. Doyere V. (2007). The effect of an intruded event on peak-interval timing in rats: isolation of a postcue effect. Behav. Processes 74, 300–310 10.1016/j.beproc.2006.11.004 17161921

[B5] Buhusi C. V. (ed.). (2003). Dopaminergic Mechanisms of Interval Timing and Attention. Boca Raton, FL: CRC Press 10.1016/j.beproc.2006.10.004

[B6] Buhusi C. V. (2012). Time-sharing in rats: effect of distracter intensity and discriminability. J. Exp. Psychol. Anim. Behav. Process. 38, 30–39 10.1037/a0026336 22122061 PMC3636330

[B7] Buhusi C. V. Meck W. H. (2000). Timing for the absence of a stimulus: the gap paradigm reversed. J. Exp. Psychol. Anim. Behav. Process. 26, 305–322 10913995 10.1037//0097-7403.26.3.305

[B8] Buhusi C. V. Meck W. H. (2002). Differential effects of methamphetamine and haloperidol on the control of an internal clock. Behav. Neurosci. 116, 291–297 10.1016/j.beproc.2006.10.004 11996314

[B9] Buhusi C. V. Meck W. H. (2005). What makes us tick? Functional and neural mechanisms of interval timing. Nat. Rev. Neurosci. 6, 755–765 10.1038/nrn1764 16163383

[B10] Buhusi C. V. Meck W. H. (2006). Interval timing with gaps and distracters: evaluation of the ambiguity, switch, and time-sharing hypotheses. J. Exp. Psychol. Anim. Behav. Process. 32, 329–338 10.1037/0097-7403.32.3.329 16834500

[B11] Buhusi C. V. Meck W. H. (2007). Effect of clozapine on interval timing and working memory for time in the peak-interval procedure with gaps. Behav. Processes 74, 159–167 10.1016/j.beproc.2006.10.004 17141425 PMC1849977

[B12] Buhusi C. V. Meck W. H. (2009a). Relative time sharing: new findings and an extension of the resource allocation model of temporal processing. Philos. Trans. R. Soc. Lond. B Biol. Sci. 364, 1875–1885 10.1098/rstb.2009.0022 19487190 PMC2685821

[B13] Buhusi C. V. Meck W. H. (2009b). Relativity theory and time perception: single or multiple clocks? PLoS ONE 4:e6268 10.1371/journal.pone.0006268 19623247 PMC2707607

[B14] Buhusi C. V. Meck W. H. (eds.). (2010). Timing Behavior. Berlin: Springer

[B15] Chiew K. S. Braver T. S. (2011). Positive affect versus reward: emotional and motivational influences on cognitive control. Front. Psychology 2:279 10.3389/fpsyg.2011.00279 22022318 PMC3196882

[B16] Church R. M. (1978). The internal clock, in Cognitive Processes in Animal Behavior, eds Hulse S. Fowler H. Honig W. (Hillsdale, NJ: Erlbaum), 277–310

[B17] Church R. M. (1984). Properties of the internal clock. Ann. N.Y. Acad. Sci. 423, 566–582 10.1111/j.1749-6632.1984.tb23459.x 6588815

[B18] Church R. M. Meck W. H. Gibbon J. (1994). Application of scalar timing theory to individual trials. J. Exp. Psychol. Anim. Behav. Process. 20, 135–155 8189184 10.1037//0097-7403.20.2.135

[B19] Coull J. T. Cheng R. K. Meck W. H. (2011). Neuroanatomical and neurochemical substrates of timing. Neuropsychopharmacology 36, 3–25 10.1038/npp.2010.113 20668434 PMC3055517

[B20] National Research Council. (1996). Guide for the Care and use of Laboratory Animals. Washington, DC: National Academies Press

[B21] Davidson R. J. Irwin W. (1999). The functional neuroanatomy of emotion and affective style. Trends Cogn. Sci. 3, 11–21 10.1016/S1364-6613(98)01265-0 10234222

[B22] Denkova E. Wong G. Dolcos S. Sung K. Wang L. Coupland N. (2010). The impact of anxiety-inducing distraction on cognitive performance: a combined brain imaging and personality investigation. PLoS ONE 5:e14150 10.1371/journal.pone.0014150 21152391 PMC2994755

[B23] Dolcos F. McCarthy G. (2006). Brain systems mediating cognitive interference by emotional distraction. J. Neurosci. 26, 2072–2079 10.1523/JNEUROSCI.5042-05.2006 16481440 PMC6674921

[B24] Droit-Volet S. Meck W. H. (2007). How emotions colour our perception of time. Trends Cogn. Sci. 11, 504–513 10.1016/j.tics.2007.09.008 18023604

[B2] DSM-IV-TR. (2000). Diagnostic and Statistical Manual of Mental Disorders, 4th Edn. Text Rev. Washington, DC: American Psychiatric Association 10.1037/a0027766

[B25] Etkin A. Egner T. Kalisch R. (2011). Emotional processing in anterior cingulate and medial prefrontal cortex. Trends Cogn. Sci. 15, 85–93 10.1016/j.tics.2010.11.004 21167765 PMC3035157

[B26] Etkin A. Wager T. D. (2007). Functional neuroimaging of anxiety: a meta-analysis of emotional processing in PTSD, social anxiety disorder, and specific phobia. Am. J. Psychiatry 164, 1476–1488 10.1176/appi.ajp.2007.07030504 17898336 PMC3318959

[B27] Gibbon J. Church R. M. Meck W. H. (1984). Scalar timing in memory. Ann. N.Y. Acad. Sci. 423, 52–77 10.1111/j.1749-6632.1984.tb23417.x 6588812

[B28] Gohier B. Ferracci L. Surguladze S. A. Lawrence E. El Hage W. Kefi M. Z. (2009). Cognitive inhibition and working memory in unipolar depression. J. Affect. Disord. 116, 100–105 10.1016/j.jad.2008.10.028 19042027

[B29] Jiao X. Pare W. P. Tejani-Butt S. M. (2006). Antidepressant drug induced alterations in binding to central dopamine transporter sites in the Wistar Kyoto rat strain. Prog. Neuropsychopharmacol. Biol. Psychiatry 30, 30–41 10.1016/j.pnpbp.2005.06.017 16091300

[B30] Johnson L. R. Hou M. Prager E. M. Ledoux J. E. (2011). Regulation of the fear network by mediators of stress: norepinephrine alters the balance between cortical and subcortical afferent excitation of the lateral amygdala. Front. Behav. Neurosci. 5:23 10.3389/fnbeh.2011.00023 21647395 PMC3102213

[B31] Kim J. Jung A. H. Byun J. Jo S. Jung M. W. (2009). Inactivation of medial prefrontal cortex impairs time interval discrimination in rats. Front. Behav. Neurosci. 3:38 10.3389/neuro.08.038.2009 19915730 PMC2776483

[B32] Ledoux J. (2007). The amygdala. Curr. Biol. 17, R868–R874 10.1016/j.cub.2007.08.005 17956742

[B33] Lejeune H. (1998). Switching or gating? The attentional challenge in cognitive models of psychological time. Behav. Processes 44, 18 10.1016/s0376-6357(98)00045-x 24896971

[B34] Macdonald C. J. Meck W. H. (2004). Systems-level integration of interval timing and reaction time. Neurosci. Biobehav. Rev. 28, 747–769 10.1016/j.neubiorev.2004.09.007 15555682

[B35] Marquardt D. W. (1963). N algorithm for least-squares estimation of nonlinear parameters. J. Soc. Ind. Appl. Math. 11, 11

[B36] Masana M. Bortolozzi A. Artigas F. (2011). Selective enhancement of mesocortical dopaminergic transmission by noradrenergic drugs: therapeutic opportunities in schizophrenia. Int. J. Neuropsychopharmacol. 14, 53–68 10.1017/S1461145710000908 20701825

[B37] Masana M. Castane A. Santana N. Bortolozzi A. Artigas F. (2012). Noradrenergic antidepressants increase cortical dopamine: potential use in augmentation strategies. Neuropharmacology 63, 675–684 10.1016/j.neuropharm.2012.05.020 22652058

[B38] Matell M. S. Bateson M. Meck W. H. (2006). Single-trials analyses demonstrate that increases in clock speed contribute to the methamphetamine-induced horizontal shifts in peak-interval timing functions. Psychopharmacology 188, 201–212 10.1007/s00213-006-0489-x 16937099

[B39] Matell M. S. King G. R. Meck W. H. (2004). Differential modulation of clock speed by the administration of intermittent versus continuous cocaine. Behav. Neurosci. 118, 150–156 10.1037/0735-7044.118.1.150 14979791

[B40] Meck W. H. Macdonald C. J. (2007). Amygdala inactivation reverses fear's ability to impair divided attention and make time stand still. Behav. Neurosci. 121, 707–720 10.1037/0735-7044.121.4.707 17663596

[B41] MED Associates. (1999). WMPC software (Version 1.15). (Computer software, St. Albans, VT).

[B42] Morrow B. A. Elsworth J. D. Rasmusson A. M. Roth R. H. (1999). The role of mesoprefrontal dopamine neurons in the acquisition and expression of conditioned fear in the rat. Neuroscience 92, 553–564 10.1016/S0306-4522(99)00014-7 10408604

[B43] Olton D. S. Wenk G. L. Church R. M. Meck W. H. (1988). Attention and the frontal cortex as examined by simultaneous temporal processing. Neuropsychologia 26, 307–318 3399046 10.1016/0028-3932(88)90083-8

[B44] Paxinos G. Watson C. (eds.). (1998). The Rat Brain. San Diego, CA: Academic Press

[B45] Penney T. B. Holder M. D. Meck W. H. (1996). Clonidine-induced antagonism of norepinephrine modulates the attentional processes involved in peak-interval timing. Exp. Clin. Psychopharmacol. 4, 10

[B46] Pessoa L. (2008). On the relationship between emotion and cognition. Nat. Rev. Neurosci. 9, 148–158 10.1038/nrn2317 18209732

[B47] Rammsayer T. H. Hennig J. Haag A. Lange N. (2001). Effects of noradrenergic activity on temporal information processing in humans. Q. J. Exp. Psychol. B 54, 247–258 10.1080/713932756 11547514

[B48] Robinson D. L. Wightman R. M. (2004). Nomifensine amplifies subsecond dopamine signals in the ventral striatum of freely-moving rats. J. Neurochem. 90, 894–903 10.1111/j.1471-4159.2004.02559.x 15287895

[B49] Rose E. J. Ebmeier K. P. (2006). Pattern of impaired working memory during major depression. J. Affect. Disord. 90, 149–161 10.1016/j.jad.2005.11.003 16364451

[B50] Rosenkranz J. A. Grace A. A. (2002). Cellular mechanisms of infralimbic and prelimbic prefrontal cortical inhibition and dopaminergic modulation of basolateral amygdala neurons *in vivo*. J. Neurosci. 22, 324–337 11756516 10.1523/JNEUROSCI.22-01-00324.2002 PMC6757602

[B51] Schirmer A. (2011). How emotions change time. Front. Integr. Neurosci. 5:58 10.3389/fnint.2011.00058 22065952 PMC3207328

[B53] Sierra-Mercado D. Padilla-Coreano N. Quirk G. J. (2011). Dissociable roles of prelimbic and infralimbic cortices, ventral hippocampus, and basolateral amygdala in the expression and extinction of conditioned fear. Neuropsychopharmacology 36, 529–538 10.1038/npp.2010.184 20962768 PMC3005957

[B54] Stevens M. C. Kiehl K. A. Pearlson G. Calhoun V. D. (2007). Functional neural circuits for mental timekeeping. Hum. Brain Mapp. 28, 394–408 10.1002/hbm.20285 16944489 PMC6871423

[B55] Swearingen J. E. Buhusi C. V. (2010). The pattern of responding in the peak-interval procedure with gaps: an individual-trials analysis. J. Exp. Psychol. Anim. Behav. Process. 36, 443–455 10.1037/a0019485 20718550 PMC2964407

[B56] Taylor K. M. Horvitz J. C. Balsam P. D. (2007). Amphetamine affects the start of responding in the peak interval timing task. Behav. Processes 74, 168–175 10.1016/j.beproc.2006.11.005 17222991

[B57] Tejani-Butt S. Kluczynski J. Pare W. P. (2003). Strain-dependent modification of behavior following antidepressant treatment. Prog. Neuropsychopharmacol. Biol. Psychiatry 27, 7–14 10.1016/S0278-5846(02)00308-1 12551720

[B58] Uylings H. B. Groenewegen H. J. Kolb B. (2003). Do rats have a prefrontal cortex? Behav. Brain Res. 146, 3–17 10.1016/j.bbr.2003.09.028 14643455

[B59] Vertes R. P. (2004). Differential projections of the infralimbic and prelimbic cortex in the rat. Synapse 51, 32–58 10.1002/syn.10279 14579424

[B60] Zakay D. (2000). Gating or switching? Gating is a better model of prospective timing (a response to ‘switching or gating?' by Lejeune)(1). Behav. Processes 52, 63–69 10.1016/S0376-6357(00)00141-8 11164674

